# Attitudes toward the Care of Children with Cancer in Saudi: An Exploratory Survey

**DOI:** 10.3390/children10040693

**Published:** 2023-04-06

**Authors:** Ghiath Alahmad

**Affiliations:** King Abdullah International Medical Research Center (KAIMRC), King Saud Bin Abdulaziz University for Health Sciences, Riyadh 11481, Saudi Arabia; ghiathalahmad@hotmail.com or alahmadgh@kaimrc.edu.sa; Tel.: +966-539-459-000

**Keywords:** pediatric oncology, care, treatment refusal, consent, assent

## Abstract

The ethical challenges of pediatric cancer care across Arab countries are not well addressed, despite medical advancements and increased awareness of children’s rights. The ethical challenges related to pediatric cancer in Saudi Arabia were investigated by surveying 400 respondents at King Abdulaziz Medical City in Riyadh, Jeddah, and Dammam, Saudi Arabia, from four groups: pediatricians, medical students, nurses, and parents of children with cancer. Respondents’ characteristics were surveyed across three outcomes: awareness of care, knowledge, and parent consent/child assent, developed from a systematic review and a qualitative analysis. A majority of respondents (89.0%) considered pediatric cancer different from adult cancer. Families considered alternative treatment, according to 64.3% of respondents, while 88.0% emphasized understanding the family’s needs and values. Furthermore, 95.8% of respondents believed physicians should offer time for pedagogy, 92.3% viewed parental consent as essential, and 94.5% thought that sufficient discussion about the plan and type of treatment should precede consent. However, child assent showed lower levels of agreement, with only 41.3% and 52.5% agreeing with getting child assent and having a discussion. Finally, 56% agreed that parents might refuse suggested treatment, while only 24.3% agreed that the child could refuse it. In all these ethical considerations, nurses and physicians showed significantly more positive results compared with other groups.

## 1. Introduction

There is no more sensitive and concerning issue to the sensibilities of most people than a child who has cancer. Statistics show that there are around 400,000 children and adolescents aged 0–19 diagnosed with cancer every year worldwide [1]. The annual incidence of pediatric cancer in Saudi Arabia is 99.83 per million people [2]. Statistics also show that pediatric cancer is the disease that causes more deaths than any other illness for children, coming in third place after motor vehicle crashes and firearm-related injuries [3]. Without a doubt, pediatric cancer evokes pity, sadness, and anger among people who are concerned about those children, especially the parents who see their child suffering from the disease’s progress and treatment consequences and are afraid of losing their child at every moment [4].

Pediatric cancer and the difficulty treating it, including dealing with its effects, place heavy burdens of responsibility on physicians, nurses, and other healthcare providers toward those children who have cancer and their families [5]. This responsibility may be affected by physicians and other healthcare providers and their points of view toward the rights of children and their families, as well as by the child’s social and economic circumstances, family situations, and society in general. Healthcare providers base their decisions on the child’s needs and behavior, so they will know precisely how to deal with a particular child. 

In the last decade, substantial and rapid medical advances in diagnosis and treatment methods have been developed. At the same time, an increase in awareness of human rights has developed, and respect for human dignity has grown. These developments reflect what has become known as the principles of biomedical ethics, especially the four well-known principles: autonomy, beneficence, non-maleficence, and justice. These human principles, first established in western philosophy, are seen nowadays as universal principles, as they are widely accepted all over the world. However, at the same time, it cannot be guaranteed that all nations will agree on all principles, details, parts, and applications. This is understandable because of various cultures and social, economic, and political developments. On the other hand, changes in the traditional view of society about the rights of children and adults contribute to the need to examine changes and developments with regard to recognizing children’s identity and autonomy. It is imperative to know in this regard that Saudi society is mainly built on strong family relationships, extended families, and tribal structure, and most importantly, that elderly people have a high level of respect [6]. Their words should always be heard and obeyed, and any behavior against them may not be tolerated.

On the other side, the families of children with cancer, after recovering from the shock of the initial diagnosis, mostly suffer from many difficult issues, especially when they see their child suffering from an exhausting disease with a poor prognosis and scary progression [7]. It is essential to recognize and understand the reality of families and their points of view, and that their beliefs and thoughts will affect the care given [7]. This understanding requires a high level of communication skills from healthcare providers. [8]

In recent decades, as a result of great economic growth, Saudi Arabia has witnessed great leaps in the development of health care . This applies to the care of children with cancer, as this specialization has witnessed great interest and rapid growth through the establishment of specialized units in many large hospitals, the qualification of doctors in the western world, and the formation of associations concerned with providing care for children with cancer, such as the Sanad Association. In addition, the difference in the cultural, social, and religious backgrounds of working doctors and nurses influences their attitudes towards caring for children with cancer.

Although the views of healthcare providers and families toward pediatric cancer have been widely explored around the world, as seen in the medical literature, the views of people in the Middle East have not been sufficiently studied. Thus, the aims of this study were to: assess the opinions of different categories of the Saudi community, including physicians, medical students, nurses, and families, toward pediatric cancer and the duties of health-care providers; and determine their attitudes and the norms toward parental consent and child assent in pediatric cancer care.

## 2. Materials and Methods

In this cross-sectional study, awareness regarding the care of children with cancer was assessed, while various concepts related to pediatric cancer and medical ethics were investigated and surveyed. The participants were questioned about their understanding and awareness regarding each subject. Study participants who had medical knowledge were specifically chosen, but with differing levels of experience. Specifically, these participants were selected to represent one of four groups: physicians, nurses, medical students, and family members of pediatric cancer patients. Each of the four study groups assessed ultimately contained 100 participants, i.e., in each category, 25% of the total of 400 participants at King Abdulaziz Medical City in Riyadh, Jeddah, and Dammam, Saudi Arabia, from June to December 2018. The sample size was determined to be similar to previous studies conducted in a similar environment [9,10]. The questionnaires were completed by three groups by themselves: physicians, nurses, and medical students, while participants from children’s families got help from research coordinators.

These participants were surveyed for demographic information, as noted below, as well as their awareness of various statements regarding the care of pediatric oncology patients. Participants’ characteristics were tested across 13 questions in three outcomes. The first four questions inquired about participants’ awareness of care. Another four questions in the second section measured the factors associated with the level of awareness of the right to know. The five questions in the third section assessed participants’ views on the factors associated with parent consent/child assent. The three outcomes—awareness of care, knowledge, and parent consent/child assent—all developed from a systematic review [11] and a qualitative analysis [12]. The questionnaires were in both Arabic and English, and their validity was assured by several experts. Test-retest reliability was ensured in a pilot study of five subjects before formal data collection commenced. Cronbach’s alpha reliability was used, and the questionnaire achieved a consistency coefficient of 0.87. Responses on a 5-point Likert scale were converted to a 3-point scale and then analyzed.

The Institutional Ethics Committee of the King Abdullah International Medical Research Center granted approval to conduct this research. Every participant provided informed consent after being informed about the research and its goals. Participants were also given the right to withdraw at any time without any negative consequences. Respect for privacy and confidentiality were assured at all stages of the research.

Data analysis was conducted using SPSS, Version 25. The demographic data of participants’ characteristics were illustrated in frequencies and percentages, and the two study outcomes were presented as percentage mean scores (PMS; ± standard deviation). A coefficient of skewness was used to assess the normality of the distribution of responses regarding the care of the child with cancer. Other normally distributed outcomes were statistically tested using the student’s t-test and one-way analysis of variance (ANOVA). For all statistical analyses, a *p*-value < 0.05 was considered significant.

## 3. Results

### 3.1. Participants’ Characteristics

The only parameter involved in the selection process was the participants’ current job title, where each category comprised 25% of the total. The participants’ ages were grouped into two main groups: those greater than 39 years of age (69.75%) and those 39 years of age and younger (30.25%). Since this research was conducted with local participants, their nationalities were noted as Saudi (69%) or various non-Saudi (31%). The majority of respondents were Muslims (84.3%), while 13.5% were Christians, and 2.2% came from other religions. Slightly more than half (55.3%) of the participants were married. The participants’ highest level of education was documented, ranging from secondary school (10.8%) to post-graduate (15.5%), with diploma or bachelor’s degree holders forming the majority (73.8%). Other sample characteristics are listed in Table 1. 

### 3.2. Attitudes on the Complexity of Pediatric Cancer

Pediatric cancer was seen as different from adult cancer and from other children’s diseases by 89.8% of the participants. Likewise, 88.0% of the participants agreed on the importance of understanding the family’s needs. However, only 64% agreed that families usually consider all alternative options in treatment (Table 2). 

The results did not show significant differences in regard to age, except for pediatric cancer being different from adult cancer, in which people above 39 years old showed slightly higher levels of agreement (91.7%) compared with people less than 39 years old (88.9%). Females showed higher levels of agreement (90.3%) than males (84.3%) in regard to the importance of considering the family’s needs and values; for other statements, no difference was noted between the two genders. A higher percentage of non-Muslim (76.2%) than Muslim (62%) participants agreed that families usually consider all alternative treatment options and the family’s needs and values. Married participants expressed a slightly higher level of agreement that pediatric cancer is different from adult cancer compared with singles (91.3% and 89.1%, respectively). No differences based on level of education were noted for the purpose of understanding the family’s needs and values, while post-graduate participants expressed significantly higher levels of agreement than those in other educational groups in regard to the statements about caring for children with cancer being different than caring for adults (100.0%); caring for children with cancer being different from caring for children with other diseases (98.4%); and the importance of alternative treatments (71.0%). Non-Saudis had higher levels of agreement compared with Saudis (73.4% compared with 60.0%, respectively) to consider alternative treatments, and 93.5% compared with 85.5% for understanding the family’s needs and values. Physicians and nurses had significantly higher levels of agreement compared with others for all statements (Table 3).

### 3.3. Attitudes toward the Right to Know

Regarding the right of the child and parents to obtain information, the results were clearly positive, with 94.5% of the participants indicating that the doctor should explain the treatment plan to the parents, and 91.3% saying the doctor should tell them whether there is a cure or a palliative treatment. However, only 52.5% of the respondents supported explaining the treatment plan to the child. The study showed a clear positive (95.8%) trend towards the responsibility of the doctor towards offering time for educating the child and his family about the illness (Table 2).

The Saudis agreed at a higher rate (92.4%) than non-Saudis (88.7%) about the necessity of giving information to the parent or child and about the responsibility of doctors to provide time for education (83.6% and 78.2%, respectively); however, the Saudis showed a lower rate regarding discussing treatment plans with parents (94.2%) and children (49.5%) compared with non-Saudis (96.0% and 59.7%, respectively). Compared to singles (91.4%), married couples agreed at a slightly higher rate regarding the necessity of informing the parents whether the treatment is curative or palliative (92.5%). The study also showed a clear positive trend among nurses in agreeing with the right of parents and children to be informed. Doctors agreed at a higher rate (up to 87.0%) on providing time for parent and child education, while patients’ families had the lowest rate (77.0%). Non-Muslims showed higher responses in regard to discussing the treatment plan with parents and children (95.2% and 71.4%, respectively) than Muslims (94.4% and 49.0%, respectively), while patients’ families got the lowest score (40.0%) for discussing the plan with children. The MA and PhD holders agreed at a higher rate regarding providing information, discussing treatment plans with parents and children, and providing time for education (Table 4).

### 3.4. Attitudes toward Parental Consent and Child Assent

The overall percentage scores of attitudes toward parental consent were significantly higher than those toward child assent (92.3% and 41.3%, respectively). There was agreement as well, but at lower percentages, about parents and children refusing treatment (56.3% and 24.3%, respectively). A fraction of 69% of participants agreed that it is acceptable for physicians to influence patients’ decisions. At the same time, age had no effect on any of the research statements, except regarding child assent, in which the participants above 39 years old showed a higher rate of agreement (50.8%) than the younger participants (37.1%); however, both females and married participants showed higher responses, especially for child assent (45.7% and 44.3%, respectively) and treatment refusal by child (26.3% and 26.2%, respectively). Detailed responses to individual awareness statements are given in Table 2 and Table 5.

Both non-Saudi and non-Muslim participants had higher levels of awareness of parental consent (96.0% and 96.8%, respectively) and agreement on parental treatment refusal (71.8% and 55.6%, respectively), as well as child assent (62.9% and 76.2%, respectively) and child treatment refusal (38.7% and 46.0%, respectively). 

Nurses, followed by physicians, had significantly higher levels of awareness on all the statements concerning parent consent (98.0% and 97.0%, respectively) and child assent (67.0% and 41.0%, respectively), except regarding the statement: “It is acceptable for the physicians to influence patients’ decisions.” Other statistically significant differences can be observed in Table 5.

## 4. Discussion

### 4.1. Care of Children with Cancer 

Children receive more compassion than adults because of their weakness and lack of ability, which explains why parents consider child cancer to be different from adult cancer. Doctors and nurses may consider pediatric cancer to be different, as children are not yet adults and the manifestations and treatment methods of the disease are different for children than for adults. However, one significant difference between children and adults is the disparity between their decision-making abilities and their ability to understand the disease, its implications, signs, and symptoms—all of which necessitate the use of simple explanations and easy-to-understand phrases when communicating with children [13,14].

There is widespread recognition that cancer is different from other diseases, including in the nature of the disease, methods of treatment, and its duration—which may extend for years. Moreover, these differences are highlighted to the fullest in the use of chemotherapy, which has effects on the whole body and leads to weakening and decreased immunity [15]. The majority of participants in this study affirmed that cancer is different from other diseases. Doctors and nurses agreed on that at a higher rate, which may be due to their understanding of the nature of cancer and the effects of treatment, especially because the long period of treatment paves the way to developing a relationship between the child and the cancer-treating team, especially nurses. This kind of relationship may be different when compared with patients who come for emergency or acute care. Moreover, but not in the least, psychological effects can be more pronounced on the surrounding people, including parents and nurses, among others [16].

Cultural and regional differences may not play a role in the treatment of cancer, which follows therapeutic protocols based on scientific principles that do not differ by culture or region. Understanding the values and needs of families, however, can contribute to alleviating suffering and may play a role in how to deal with them. In addition, because cancer often requires several years of treatment, it seems more important to provide adequate palliative care [17]. A lack of understanding of the family’s social and cultural background can lead either to a child’s failure to continue with the treatment plan or to resort to alternative methods. This was reflected in the study results, where more than 85% agreed on the importance of taking the social aspects into account, a finding that is supported by other studies [18]. Nurses and doctors were the groups that expressed the importance of this the most, as this is the core of good performance in their jobs.

In order to consider and understand cultural differences, it is important to remove language barriers that may cause a lack of proper understanding of the patient’s concerns [19]. Therefore, it seems appropriate that nurses and doctors acknowledge that patients resort to alternative methods of treatment, which seems to be a common practice even in other countries. Although these alternative methods are not identified in our questionnaire, they may include Quran recitation, prayer, folk medicine, etc.

Being aware of patients’ access to alternative methods and what these methods are may allow the treatment team to assess and treat the condition, while providing appropriate recommendations to patients and their families. Although we cannot assess the extent to which patients resort to alternative means, 65% of our respondents reported being aware that families often seek alternative therapies; however, the health-care providers do not usually know the exact nature of these alternative therapies. Other studies showed that parents used to discuss the use of alternative therapies with their children’s physicians [20,21,22].

### 4.2. Communicating Information to Parents

A large majority, up to 90%, agreed on the importance of telling parents whether the child would receive the cancer-curing treatment or the palliative treatment only. This is consistent with the patient’s right (and his or her parents’ in the case of a child) to obtain adequate and correct information regarding their illness. Having adequate information helps parents build their plans and expectations and balance their options, while preventing them from building false dreams and false expectations that may be disappointing [23].

Professional strategies on how to communicate with and inform the family must be followed. In this regard, it is helpful for the treatment team to include social specialists, who must be trained, tolerant, and patient [24]. The quality and methods of communication with parents play an important role in the treatment of the child and have the potential to raise morale. It has been observed that the child and the parents show greater commitment to treatment when communication is better [8,25]. Knowing that the disease is curable, and that effective treatment is available but not affordable in all cases presents a moral challenge that requires highly effective communication.

The support provided by the medical team remains extremely important and includes providing accurate and adequate information. Moreover, the conversation with the patient’s parents should not be limited only to sharing information but also to building confidence. Support, however, may lead to influence, as accepted by many of our respondents and recognized by other authors [26].

### 4.3. Teaching the Patient

Child cancer is still seen as a serious disease with a bad reputation and is associated with death, regardless of the great advances in diagnosis and treatment. This is especially understandable given the history of the disease, which places a responsibility on the medical staff to educate and enlighten parents and increase their knowledge about treatment methods and disease prognosis. This will not be limited to providing them with scientific information only but also techniques of psychosocial treatments, including cognitive behavioral therapy, relaxation therapy, and art therapy [27,28]. Therefore, more than 90% of the participants in the research supported psychosocial intervention; this was especially true among doctors, who showed a clearer commitment to psychosocial intervention, while there was no difference in this preference between the other demographic groups.

Since doctors and nurses use a variety of educational methods and procedures, the therapeutic teams need to include people who specialize in patient education. Patient and family education is becoming increasingly important, especially for the parents’ role in the treatment plan, including home-based treatment away from the hospital. At the same time, some patients’ families are still not aware of the importance of education in improving care quality, or they believe that the central role of physicians is not to educate patients and their parents but to offer them the best available treatment.

### 4.4. Informed Consent

More than 90% of participants expressed the need to get informed consent, which reflects the importance of this as the moral pillar of medical practice and as the moral awareness that no action can be taken without prior consent. Nurses supported informed consent at the highest percentages (i.e., 98%), followed by doctors at 97%. The fact that the level of support was less than 100% can be explained by the need to act quickly in some emergency cases that require direct intervention. The percentage of students supporting informed consent was somewhat lower, yet still at 86%, which may indicate the need for training courses.

The way consent is obtained is no less important than obtaining the consent itself. It is important to provide sufficient time for understanding and decision making. This should be expressed in simple language, without coercion or compulsion, and with the identification of alternative therapeutic aspects and possibilities, as well as the side effects of any drug used. Verbal consent alone is not enough; it must be in writing, and moreover, there must be a willingness to answer questions that are on the minds of parents. Furthermore, the use of a single consent may not be sufficient, especially when the treatment plan is completed, or a new treatment plan is to be implemented.

The study showed an overwhelming majority of 94.5% of all participants, and 98% of doctors and 97% of nurses agreed with the importance of discussing the treatment plan with parents. This reflects the importance of moral commitment in practice, the degree of knowledge of ethical principles, and the acknowledgement of the ability of parents to know how to make the right decisions. Given the great role parents play in the treatment of children and the difficulty of making decisions, access to information is necessary before parents are asked to accept any action. Sharing information and helping parents understand is so important that, when necessary, specialists should be used to overcome the difficulties, taking into account that parents can vary in their abilities. In some cases, additional challenges may arise when one parent wishes to conceal information about the other when he or she is the most emotional or ill.

### 4.5. Children and Assent

More than half of respondents considered the assent of children to be important. This level of support for the involvement of children, especially given that the talk was general, reflects the desire to involve children in making decisions regarding their health. Women were more likely to accept children being involved in assent, as were older and more educated persons, while marital status did not affect opinions. Nurses, followed closely by doctors, expressed the highest levels of agreement with the statement about the importance of using child assent.

The process of obtaining assent from a sick child may be difficult due to factors such as the unwillingness of parents to inform the child about his or her illness out of fear of affecting the child’s psychological state [29]. Although the child’s assent is linked to the child’s mental ability and capacity for judgment, often a certain minimum age is set, although it is not the same age in all countries.

The assent of the child is still important, even if it is not fully understood and treated effectively, which explains the agreement of more than half of the participants that the child’s assent is important. This also reflects the fact that childhood cancer is a family disease, and any decision made should involve the participation of the parents and the child [30].

More than 60% of the participants supported the importance of talking with the child prior to assent, which is close to the level of agreement with the importance of assent itself. Nurses and doctors were more inclined to conduct discussions, which could be interpreted as the result of clinical experience and the nature of their work that places them in direct contact with children, giving them opportunities to develop relationships with their child patients. The same justification also applies to participants over the age of 39, who have shown greater acceptance for the child’s assent compared to younger groups, such as students. 

Since most participating nurses were non-Muslims and non-Saudi, the three categories—nurses, non-Muslims, and non-Saudi—were similar in showing a higher rate of agreement regarding the importance of using children’s assent and discussing the treatment plan with children.

The low rate of expressed interest by the patients’ families regarding the treatment plan discussion and the importance of children’s assent may reflect parental desire to protect their children from any psychological pain. Moreover, in many patients’ families, the parents will make the final decision, while their children may have no fundamental role. 

On the other hand, the discussion with children explains why we see other authors going further in encouraging child participation in shared decision making [31]. There is no doubt that such discussions should be conducted in a professional manner that takes into account the child’s state and mental ability, and in the presence of at least one family member.

### 4.6. Parents’ and Children’s Treatment Refusal 

More than 50% of respondents agreed that parents had the right to refuse treatment, while less than a quarter (24.3%) agreed with the child’s right to do so. The respondent parents had the lowest level of agreement with the right to refuse treatment, while the highest levels of agreement were on the part of nurses and doctors. These high percentages clearly reflect that Saudi society still typically defers to the opinions of doctors and nurses, whom they trust and consider the most knowledgeable. This trust in physicians and nurses is also supported by other results in this study, which show that Saudi participants also showed the lowest level of agreement with the importance of obtaining parental consent and child assent and the importance of understanding a family’s needs and values.

The right of parents and children to refuse treatment presents a moral dilemma in that respecting their autonomy in free decision-making may have negative effects on the child patient, especially when refusal of treatment causes their health to deteriorate. In addition, the rights of patients and parents to refuse treatment place a moral responsibility on doctors and nurses to provide enough information and explanation in an attempt to convince parents and children of the best treatment options in order to have better protection from harm.

Rejecting treatment can manifest in different forms and degrees, including complete refusal of treatment or refusal to complete a course of treatment. It may be a rejection of a specific part of the treatment, such as chemotherapy. Rejection may also not be complete rejection but the desire to replace it with another drug or change the method of administering the drug. Refusal of medical treatment may also accompany resorting to non-traditional treatments such as prayer and recitation.

The reason for treatment refusal is often a lack of full understanding of the disease and its treatment. Treatment decisions can be further complicated by several factors: personal, family, psychological, financial, and others. Sometimes the experience of severe side effects and dissatisfaction with the efficacy of the treatment can lead to such refusal [32].

If parents refuse to treat their cancerous child and deem the treatment harmful, then permitting it becomes morally problematic. The adult’s right to refuse their own treatment may be acceptable, but it may not be morally correct if the refusal is on behalf of a child who is vulnerable, especially when the decision may cause harm to the child, such as when refusing a lifesaving medicine.

### 4.7. Limitations

A relatively small sample size can be considered one of the limitations of this study. The sample size was determined to be similar to previous studies, which seems not to be the best method to determine it. In regard to the group of children’s families, we did not investigate the degree of relationship between respondent relatives, nor did we consider family size or whether they were independent or extended families. Moreover, using two languages in the questionnaires (Arabic for family members and English for other groups) and using two methods to collect the questionnaires—self-completed and with help from research coordinators—may have an impact on the answers. The results may be more clearly generalizable in Arabic-speaking countries compared with countries that have different ethos and cultures.

## 5. Conclusions

Caring for patients with cancer, especially children, has always been accompanied by many ethical challenges. However, awareness can help doctors, nurses, parents, and child cancer patients deal with ethical challenges in order to reach the best treatment options, as well as the best practices for consent, assent, or treatment rejection.

Pediatric cancer is seen as different from adult cancer and from other children’s diseases, and it is important to understand the family’s needs, and that families usually consider all alternative options in treatment, such as folk medicine, Quran recitation, and prayer, which can be seen as a reflection of Saudi ethos.

The participants agree on children’s rights and the fact that parents need to be informed regarding the treatment plan, including whether it is a cure or a palliative treatment. Moreover, the study showed a clear positive trend toward doctors’ responsibility to offer time for educating the child and their family about the illness. This may reflect that paternalism is still strong in Saudi society.

In the consent section, it was noted that parents and students expressed less interest in consent, reflecting the importance of organizing training courses for these two groups as well as providing psychological support for parents, which is playing an increasing role in the current Saudi medical system nowadays. It would be a good idea to include ethics training in the teaching and training syllabuses of the medical curriculum for children, including those with cancer.

## Figures and Tables

**Table 1:** Participants’ characteristics.

	n (%)
**Sex**	
Male	154 (38.5%)
Female	246 (61.5%)
**Age (years)**	
20 or less	2 (0.5%)
20–29	184 (46.0%)
30–39	93 (23.2%)
40–49	85 (21.3%)
50–59	32 (8.0%)
60 or above	4 (1.0%)
Mean ± Standard deviation	34.3 ± 10.3
**Marital status**	
Married	221 (55.3%)
Single	162 (40.5%)
Divorced	8 (2.0%)
Widowed	9 (2.3%)
**Education level**	
Secondary School	43 (10.8%)
Undergraduate	294 (73.8%)
Postgraduate	63 (15.5%)
**Religion**	
Muslim	337 (84.3%)
Non-Muslim	63 (15.7%)
Christian	54 (13.5)
Not specified	9 (2.2)
**Nationality**	
Saudi	276 (69.0%)
Non-Saudi	124 (31.0%)
Jordan	4
Lebanon	1
Syria	2
Palestine	5
Bahrain	1
Egypt	7
Malaysia	7
New Zealand	1
Pakistan	3
Filipino	60
Sudan	9
Not specified	26
**Current job**	
Physician	100 (25%)
Medical Student	100 (25%)
Nurse	100 (25%)
Patient’s family	100 (25%)

n: frequency, (%): percentage.

**Table 2:** Responses to various awareness statements.

	Strongly Agree/Agree n (%)	Neutral n (%)	Disagree/Strongly Disagree n (%)
**Perceptions about pediatric cancer**			
Caring for children with cancer is different from caring for adults with cancer.	359 (89.8%)	28 (7.0%)	13 (3.3%)
Caring for children with cancer is different from caring for children with other diseases.	357 (89.3%)	35 (8.8%)	8 (2.0%)
Families usually consider all alternative treatment options.	257 (64.3%)	106 (26.5%)	37 (9.3%)
It is important to understand a family’s needs and values.	352 (88.0%)	42 (10.5%)	6 (1.5%)
**Patients’ rights to know**			
Treating physicians should inform patients if the treatment is curative or palliative.	365 (91.3%)	29 (7.2)	6 (1.5%)
Physicians should offer time for pedagogy with parents and patients.	383 (95.8%)	17 (4.3%)	0 (0.0%)
Physicians should discuss the treatment plan with parents.	378 (94.5%)	21 (2.3%)	1 (0.3%)
Physicians should discuss the treatment plan with the child.	210 (52.5%)	125 (31.3%)	65 (16.3%)
**Patient consent/Child assent**			
Informed consent should be collected.	369 (92.3%)	22 (5.5%)	9 (2.3%)
It is accepted for physicians to influence patients’ decisions.	276 (69%)	91 (22.8%)	33 (8.3%)
The parents can refuse any kind of treatment.	225 (56.3%)	94 (23.5%)	81 (20.3%)
Assent should be collected.	165 (41.3%)	142 (35.5%)	93 (23.3%)
The child can refuse any kind of treatment.	97 (24.3%)	142 (35.5%)	161 (40.3%)

n: frequency, (%): percentage.

**Table 3:** Awareness of pediatric cancer care across sample characteristics.

	Caring for Children with Cancer Is Different than Caring for Adults with Cancer n (%)	Caring for Children with Cancer Is Different from Caring for Children with other Diseases n (%)	Families Usually Consider All Alternative Treatment Options n (%)	It Is Important to Understand All of the Family’s Needs and Values n (%)
**Age: n** ≤39: 279 >39: 121	249 (88.9%) 110 (91.7%)	251 (89.6%) 106 (88.3%)	180 (64.3%) 77 (64.2%)	246 (87.9%) 106 (88.3%)
	*p* = 0.201	*p* = 0.812	*p* = 0.737	*p* = 0.963
**Sex** Male Female	140 (91.5%) 219 (88.7%)	138 (90.2%) 219 (88.7%)	90 (58.8%) 167 (67.6%)	129 (84.3%) 223 (90.3%)
	*p* = 0.490	*p* = 0.878	*p* = 0.136	*p* = 0.203
**Nationality** Saudi Non-Saudi	243 (88.4%) 115 (92.7%)	244 (88.7%) 112 (90.3%)	165 (60.0%) 91 (73.4%)	235 (85.5%) 116 (93.5%)
	*p* = 0.409	*p* = 0.291	*p* = 0.035	*p* = 0.046
**Religion** Muslim Non-Muslim	302 (89.6%) 57 (90.5%)	304 (90.2%) 53 (84.1%)	209 (62.0%) 48 (76.2%)	294 (87.2%) 58 (92.1%)
	*p* = 0.975	*p* = 0.170	*p* = 0.031	*p* = 0.421
**Marital status** Single Married	197 (89.1%) 146 (91.3%)	202 (91.4%) 141 (88.1%)	149 (67.4%) 98 (61.3%)	196 (88.7%) 139 (86.9%)
	*p* = 0.596	*p* = 0.087	*p* = 0.002	*p* = 0.996
**Current job** Physician Medical student Nurse Patient’s family	94 (94.0%) 91 (91.0%) 90 (90.0%) 84 (84.0%)	99 (99.0%) 86 (86.0%) 86 (86.0%) 86 (86.0%)	75 (75.0%) 55 (55.0%) 79 (79.0%) 48 (48.0%)	96 (96.0%) 80 (80.0%) 93 (93.0%) 83 (83.0%)
	*p* = 0.368	*p* = 0.021	*p* < 0.001 *	*p* = 0.007
**Education level** Secondary school Undergraduate Postgraduate	33 (76.7%) 264 (89.5%) 62 (100.0%)	35 (81.4%) 261 (88.5%) 61 (98.4%)	25 (58.1%) 188 (63.7%) 44 (71.0%)	38 (88.4%) 257 (87.1%) 57 (91.9%)
	*p* < 0.001 *	*p* = 0.001 *	*p* = 0.042	*p* = 0.733

n: frequency, *p* = *p*-value, * = statistically significant at <0.05.

**Table 4:** Awareness of patients’ rights to know.

	Treating Physicians Should Inform Patients If the Treatment Is Curative or Palliative	Physicians Should Offer Time for Pedagogy with Parents and Patient	Physicians Should Discuss Treatment Plans with Parents	Physicians Should Discuss Treatment Plans with Children
**Age: n** ≤39: 279 >39: 121	258 (92.1%) 107 (89.2%)	270 (96.4%) 113 (94.2%)	268 (95.7%) 110 (91.7%)	148 (52.9%) 62 (51.7%)
	*p* = 0.058	*p* = 0.304	*p* = 0.127	*p* = 0.222
**Sex** Male Female	139 (90.8%) 226 (91.5%)	127 (83.0%) 201 (81.4%)	142 (92.8%) 236 (95.5%)	79 (51.6%) 131 (53.0%)
	*p* = 0.911	*p* = 0.917	*p* = 0.291 *	*p* = 0.325
**Nationality** Saudi Non-Saudi	254 (92.4%) 110 (88.7%)	230 (83.6%) 97 (78.2%)	259 (94.2%) 119 (96.0%)	136 (49.5%) 74 (59.7%)
	*p* = 0.033	*p* = 0.415	*p* = 0.460	*p* = 0.104
**Religion** Muslim Non-Muslim	308 (91.4%) 57 (90.5%)	280 (83.1%) 48 (76.2%)	318 (94.4%) 60 (95.2%)	165 (49.0%) 45 (71.4%)
	*p* = 0.436	*p* = 0.073	*p* = 0.894	*p* = 0.004 *
**Marital status** Single Married	202 (91.4%) 148 (92.5%)	177 (80.1%) 136 (85.0%)	209 (94.6%) 152 (95.0%)	116 (52.5%) 84 (52.5%)
	*p* = 0.055	*p* = 0.619	*p* = 0.372	*p* = 0.427
**Current job** Physician Medical student Nurse Patient’s family	93 (93.0%) 91 (91.0%) 91 (91.0%) 90 (90.0%)	87 (87.0%) 86 (86.0%) 78 (78.0%) 77 (77.0%)	98 (98.0%) 93 (93.0%) 97 (97.0%) 90 (90.0%)	58 (58.0%) 50 (50.0%) 62 (62.0%) 40 (40.0%)
	*p* = 0.252	*p* = 0.031	*p* = 0.139	*p* < 0.001 *
**Education level** Secondary school Undergraduate Postgraduate	38 (88.4%) 271 (91.9%) 56 (90.3%)	32 (74.4%) 243 (82.4%) 53 (85.5%)	39 (90.7%) 279 (94.6%) 60 (96.8%)	18 (41.9%) 156 (52.9%) 36 (58.1%)
	*p* = 0.952	*p* = 0.088	*p* = 0.681	*p* = 0.012

n: frequency, *p* = *p*-value, * = statistically significant at <0.05.

**Table 5:** Awareness toward parental consent and child assent across sample characteristics.

	Parent’s Consent Should Be Collected Prior to the Start of Treatment	Child’s Assent Should Be Collected Prior to the Start of Treatment	It Is Acceptable for Physicians to Influence Patients’ Decisions	The Parents Can Refuse Any Kind of Treatment	The Child Can Refuse Any Kind of Treatment
**Age: n** ≤39: 279 >39: 121	258 (92.1%) 111 (92.5%)	104 (37.1%) 61 (50.8%)	189 (67.5%) 87 (72.5%)	158 (56.4%) 67 (55.8%)	64 (22.9%) 33 (27.5%)
	*p* = 0.376	*p* = 0.010	*p* = 0.448	*p* = 0.982	*p* = 0.390
**Sex** Male Female	139 (90.8%) 230 (93.1%)	52 (34.0%) 113 (45.7%)	113 (73.9%) 163 (66.0%)	86 (56.2%) 139 (56.3%)	32 (20.9%) 65 (26.3%)
	*p* = 0.206	*p* < 0.003 *	*p* = 0.157	*p* = 0.647	*p* = 0.089
**Nationality** Saudi Non Saudi	249 (90.5%) 119 (96.0%)	87 (31.6%) 78 (62.9%)	198 (72.0%) 78 (62.9%)	135 (49.1%) 89 (71.8%)	49 (17.8%) 48 (38.7%)
	*p* = 0.161	*p* < 0.001 *	*p* = 0.031	*p* < 0.001 *	*p* < 0.001 *
**Religion** Muslim Non-Muslim	308 (91.4%) 61 (96.8%)	117 (34.7%) 48 (76.2%)	241 (71.5%) 35 (55.6%)	179 (53.1%) 46 (73.0%)	68 (20.2%) 29 (46.0%)
	*p* = 0.274	*p* < 0.001 *	*p* = 0.000	*p* = 0.004*	*p* < 0.001 *
**Marital status** Married Single	210 (95.0%) 14,490.0%)	98 (44.3%) 55 (34.4%)	157 (71.0%) 105 (65.6%)	119 (53.8%) 94 (58.8%)	58 (26.2%) 33 (20.6%)
	*p* = 0.017	*p* = 0.016	*p* = 0.824	*p* = 0.437	*p* = 0.901
**Current job** Physician Medical student Nurse Patient’s family	98 (98.0%) 86 (86.0%) 97 (97.0%) 88 (88.0%)	41 (41.0%) 25 (25.0%) 67 (67.0%) 32 (32.0%)	72 (72.0%) 71 (71.0%) 62 (62.0%) 71 (71.0%)	60 (60.0%) 55 (55.0%) 74 (74.0%) 36 (36.0%)	21 (21.0%) 14 (14.0%) 43 (43.0%) 19 (19.0%)
	*p* = 0.006	*p* < 0.001 *	*p* = 0.016	*p* = 0.001 *	*p* < 0.001 *
**Education** Secondary school Undergraduate Postgraduate	38 (88.4%) 271 (91.9%) 60 (96.8%)	15 (34.9%) 124 (42.0%) 26 (41.9%)	28 (65.1%) 199 (67.5%) 49 (79.0%)	16 (37.2%) 170 (57.6%) 39 (62.9%)	8 (18.6%) 76 (25.8%) 13 (21.0%)
	*p* = 0.470	*p* = 0.014	*p* = 0.037	*p* = 0.001	*p* = 0.180

n: frequency, *p* = *p*-value, * = statistically significant at <0.05.

## Data Availability

Parts of the datasets generated during the study are available from the corresponding author upon reasonable request.
